# Transcriptomic and Metabolomic Insights into the Effects of Arbuscular Mycorrhizal Fungi on Root Vegetative Growth and Saline–Alkali Stress Response in Oat (*Avena sativa* L.)

**DOI:** 10.3390/jof11080587

**Published:** 2025-08-09

**Authors:** Xingzhe Wang, Xiaodan Ma, Senyuan Wang, Peng Zhang, Lu Sun, Zhenyu Jia, Yuehua Zhang, Qiuli Bao, Yuying Bao, Jie Wei

**Affiliations:** 1 Key Laboratory of Forage and Endemic Crop Biotechnology, Ministry of Education, School of Life Sciences, Inner Mongolia University, Hohhot 010010, China; starwxz666@163.com (X.W.);; 2 Inner Mongolia Engineering Technology Research Center of Germplasm Resources Conservation and Utilization, Inner Mongolia University, Hohhot 010010, China; 3 Key Laboratory of Cultivated Land Capability Conservation and Improvement in Agro-Pastoral Ecotone, Ministry of Agriculture and Rural Affairs of the People’s Republic of China, Hohhot 010010, China; 4 Vocational and Technical College, Inner Mongolia Agricultural University, Baotou 014109, China

**Keywords:** AMF, mycorrhizal symbiosis, growth promotion, abiotic stress tolerance, multi-omics analysis

## Abstract

Soil salinization limits the growth of agricultural crops in the world, requiring the use of methods to increase the tolerance of agricultural crops to salinity–alkali stress. Arbuscular mycorrhizal fungi (AMF) enhance plant stress adaptation through symbiosis and offer a promising strategy for remediation. However, in non-model crops such as oat (*Avena sativa* L.), research has mainly focused on physiological assessments, while the key genes and metabolic pathways involved in AMF-mediated growth and saline–alkali tolerance remain unclear. In this study, we employed integrated multi-omics and physiological analyses to explore the regulatory mechanisms of AMF in oats under normal and saline–alkali stress. The results indicated that AMF symbiosis significantly promoted oat growth and physiological performance under both normal and saline–alkali stress conditions. Compared to the non-inoculated group under normal conditions, AMF increased plant height and biomass by 8.5% and 15.3%, respectively. Under saline–alkali stress, AMF enhanced SPAD value and relative water content by 16.7% and 7.3%, reduced MDA content by 35.8%, increased soluble protein by 21.8%, and decreased proline by 13.3%. In addition, antioxidant enzyme activities (SOD, POD, and CAT) were elevated by 18.4%, 18.2%, and 14.8%, respectively. Transcriptomic analysis revealed that AMF colonization under saline–alkali stress induced about twice as many differentially expressed genes (DEGs) as under non-saline–alkali stressed conditions. These DEGs were primarily associated with Environmental Information Processing, Genetic Information Processing, and Metabolic Processes. According to metabolomic analysis, a total of 573 metabolites were identified across treatments, mainly comprising lipids (29.3%), organic compounds (36.8%), and secondary metabolites (21.5%). Integrated multi-omics analysis indicated that AMF optimized energy utilization and antioxidant defense by enhancing phenylpropanoid biosynthesis and amino acid metabolism pathways. This study provides new insights into how AMF may enhance oat growth and tolerance to saline–alkali stress.

## 1. Introduction

With the ongoing impacts of global climate change and unsustainable irrigation and cultivation practices, soil salinization has become increasingly severe, posing a significant threat to agricultural production and food security [1]. Currently, about 20% of the world’s arable land and nearly one-third of irrigated areas are impacted by different levels of saline–alkali stress [2]. This stress disrupts crop water homeostasis and nutrient acquisition while inducing secondary damages, such as osmotic imbalance, ion toxicity, and oxidative damage [3]. The problem is particularly acute in arid and semi-arid regions, where salinization not only leads to substantial crop yield losses but also destabilizes soil microbial communities, creating a cycle of “low productivity and land degradation.” Although common agronomic practices such as optimized irrigation, soil amendments, and crop rotation can temporarily mitigate surface symptoms of saline–alkali stress, they often involve high input costs, provide only short-term relief, and are insufficient to fundamentally restore the stress resilience of the soil–crop system [4,5]. Therefore, developing effective strategies to enhance crop tolerance to saline–alkali environments has become a central challenge in agricultural science.

As an important dual-purpose crop for grain and forage, Oat (*Avena sativa* L.) is widely cultivated across temperate and subarctic regions due to its strong stress tolerance and adaptability [6]. However, oat growth and development are severely impaired under high salinity–alkalinity conditions, characterized by leaf chlorosis, poor root development, impaired nutrient uptake, and disruptions in ion homeostasis [7,8]. Therefore, enhancing its tolerance remains a critical challenge. Against this backdrop, arbuscular mycorrhizal fungi (AMF), through their symbiotic mechanisms, offer a promising strategy to enhance plant resilience and help surpass the natural saline–alkali tolerance thresholds [9].

As key members of the soil microbiome, AMF form symbiotic associations with the roots of most terrestrial plants, establishing an extensive hyphal network that penetrates root cells and creates a pathway for nutrient exchange. This symbiotic system not only directly expands the plant’s root absorption capacity, improving water and nutrient acquisition efficiency, but also enhances host stress tolerance through multi-level regulatory mechanisms [10,11,12]. AMF can activate calcium signaling, abscisic acid (ABA), and ethylene pathways, systematically inducing the expression of stress-responsive genes, ion transporters, and osmotic adjustment-related genes. Additionally, AMF facilitate the biosynthesis of osmolytes, including proline, betaine, and soluble sugars, while also enhancing antioxidant enzyme activities to mitigate oxidative stress [13,14,15,16]. Studies have also shown that AMF modulate the accumulation of secondary metabolites, including flavonoids and phenolics, and optimize carbon and nitrogen metabolism, thereby improving energy and nutrient allocation [17,18]. These mechanisms have been demonstrated to significantly enhance saline–alkali tolerance, growth performance, and biomass accumulation in crops such as maize [19], rice [20], and tomato [21].

However, while extensive research has established the efficacy of AMF in improving stress resistance in model crops, studies on non-model crops such as oats remain largely confined to physiological evaluations [22,23]. The coordinated expression of key genes and the role of critical metabolites underlying AMF-mediated growth promotion and stress tolerance in oats remain poorly understood and warrant further investigation.

In recent years, transcriptomics and metabolomics have become core tools for unraveling gene regulatory networks and metabolic reprogramming in complex plant biological systems [24]. Based on this, the present study focuses on oat to elucidate how AMF symbiosis promotes host growth and enhances saline–alkali stress tolerance, which serves as the central hypothesis of this study. Accordingly, the objectives of this study are as follows: (1) to evaluate, through physiological and biochemical analyses, the regulatory effects of AMF on oat growth, antioxidant system activity, and osmotic adjustment capacity, and to assess their roles in promoting growth and enhancing stress resistance under saline–alkali conditions; (2) to perform transcriptomic profiling to identify AMF-regulated gene expression patterns associated with growth promotion and saline–alkali tolerance; (3) to conduct metabolomic analysis to characterize the dynamic changes in key metabolites in oat tissues and highlight features of AMF-driven metabolic reprogramming; and (4) to integrate multi-omics association analyses to elucidate the coordinated regulation between core genes and metabolic pathways under AMF symbiosis. These findings not only provide molecular and physiological evidence supporting the application of AMF to enhance oat growth and stress resilience but also offer valuable insights for harnessing microbial resources in saline–alkali agricultural ecosystems.

## 2. Materials and Methods

### 2.1. Plant Materials and AMF Inoculation

Oat (*Avena sativa* L.) was used as the plant material in this study. Four treatment groups were established: non-mycorrhizal plants under normal conditions (NM), mycorrhizal plants under normal conditions (AM), non-mycorrhizal plants under saline–alkali stress (NMS), and mycorrhizal plants under saline–alkali stress (AMS). The plastic pots used had a top diameter of 20 cm, a bottom diameter of 16 cm, and a height of 15 cm. Each pot was sown with 14 seeds, with a germination rate of 92%. During the seedling stage, seedlings with uniform growth were selected and thinned, leaving 7 plants per pot for the subsequent experiments. Each treatment group consisted of 10 pots.

For the mycorrhizal treatments, a commercial inoculum of *Rhizophagus intraradices* [25] (provided by Nanjing Cuijingyuan Biotechnology Co., Ltd., Nanjing, China) was used. The inoculum was a powder formulation consisting mainly of *R. intraradices* spores (with a viable spore density of no fewer than 70 spores per gram) mixed with an inert dispersant (attapulgite clay). To ensure early establishment of symbiosis, seeds were first coated with the AMF inoculum by gently mixing them with a small amount of sterile water and the inoculum, forming a thin, uniform layer that promoted close contact between the fungal propagules and the seeds during germination. In addition, 10 g of the same inoculum was thoroughly mixed with the potting substrate prior to sowing to further facilitate root colonization. To control for any effects of the dispersant or trace nutrients, sterilized inoculum (autoclaved at 121 °C for 30 min) was applied at the same rate and in the same manner to the non-mycorrhizal controls. This ensured that any observed differences were due to active AMF rather than nutrient contributions from the inoculum.

### 2.2. Growth Conditions and Stress Treatments

The growth substrate consisted of a 2:3 (*v*/*v*) mixture of field soil and river sand, with the sand primarily composed of quartz and an average particle size of approximately 0.5 mm. The mixture was sterilized by autoclaving at 121 °C for 30 min on two consecutive days to ensure thorough sterilization. The basic physicochemical properties of the substrate after mixing were as follows: pH 8.1, EC 1.3 dS/m, organic matter content 7.4 g·kg^−1^, total nitrogen 0.85 g·kg^−1^, alkali-hydrolyzable nitrogen 68.2 mg·kg^−1^, available phosphorus 8.6 mg·kg^−1^, and available potassium 112.5 mg·kg^−1^.

Plants were grown under greenhouse conditions with day/night temperatures controlled at 30 °C/20 °C. The photoperiod of 12 h per day was maintained under natural light supplemented with full-spectrum LED lamps (400–700 nm) at an approximate light intensity of 300 μmol·m^−2^·s^−1^ PPFD. Soil moisture was maintained at 60–70% field capacity. At the tillering stage, NMS and AMS plants were irrigated with a saline–alkali solution (NaCl:Na_2_SO_4_:NaHCO_3_:Na_2_CO_3_ = 1:9:9:1) [15] at 150 mmol·L^−1^ for 7 days (Figure S1A). Subsequently, the pH and EC of the treated soil were measured at 8.9 and 5.2 dS/m, respectively. Finally, Shoots and roots were harvested separately, immediately frozen in liquid nitrogen, and stored at −80 °C for further analysis.

### 2.3. Measurement of Phenotypic and Physiological Parameters

Phenotypic and physiological parameters measured included leaf length, root length, plant height and stem diameter, which were assessed using a ruler or caliper. Biomass was evaluated based on fresh weight. Chlorophyll content was estimated by recording Soil–Plant Analysis Development (SPAD) values with a portable chlorophyll meter (SPAD-502, Konica Minolta Co., Ltd., Tokyo, Japan). Relative water content (RWC) was calculated as RWC (%) = (FW − DW)/(TW − DW) × 100%. The dry weight (DW) was determined after drying the samples at 70 °C for 48 h until a constant weight was achieved. Relative electrical conductivity (REC) was measured with a conductivity meter. All physiological indices, including malondialdehyde (MDA), proline (Pro), soluble sugars, soluble proteins, and antioxidant enzyme activities (SOD, POD, CAT), were determined using commercial assay kits (Shanghai YouXuan Biotechnology Co., Ltd., Shanghai, China) according to the manufacturer’s protocols. For each treatment, three pots (*n* = 3) were randomly selected as independent biological replicates. From each pot, samples from three plants were randomly collected and pooled to form a composite sample.

### 2.4. Measurement of Arbuscular Mycorrhizal Fungi Colonization Rate

Root samples were washed and cut into 1 cm segments, then cleared in 10% (*w*/*v*) KOH at 90 °C for 1 h. After cooling, segments were rinsed with distilled water, acidified in 2% (*v*/*v*) HCl for 30 min, and rinsed 3–5 times. Finally, roots were stained with 0.05% (*w*/*v*) trypan blue in a lactic acid–glycerol–water (1:1:1, *v*/*v*/*v*) mixture at 90 °C for 20 min. Finally, excess stain was removed by destaining in lactic acid–glycerol solution. After decolorization, 30–50 root segments were randomly selected per pot. For each treatment, three pots were randomly selected as independent biological replicates. The root segments were then spread on a microscope slide with a drop of 30% glycerol and a coverslip. Samples were observed under an optical microscope (Leica DM5500B, Leica Microsystems GmbH, Wetzlar, Germany), and the colonization rate of AMF (Figure S1B) was calculated using the root segment observation method combined with the grid cross method [26]. In the AM group, colonization occurred at a rate of 36%, while the AMS group exhibited a rate of 28%, with a significant difference between the two treatments (*p* < 0.05). Meanwhile, root samples from NM and NMS treatments were examined microscopically using the same methods and sample numbers as AM and AMS, confirming no AMF colonization, with a colonization rate of 0% (Figure S1C).

### 2.5. Transcriptome Sequencing and Data Analysis

Three pots per treatment (*n* = 3) were randomly selected as independent biological replicates. From each pot, roots from three plants were randomly collected and pooled to form a composite sample. Total RNA was extracted from oat roots using TRIzol reagent (Thermo Fisher Scientific Inc., Waltham, MA, USA), and its concentration and purity were measured with a NanoDrop spectrophotometer (Thermo Fisher Scientific Inc., Waltham, MA, USA). Sequencing libraries were prepared from 3 μg RNA per sample using the TruSeq RNA Library Prep Kit (llumina Inc., San Diego, CA, USA) following the manufacturer’s protocol. Library quality was assessed with an Agilent 2100 Bioanalyzer (Agilent Technologies Inc., Santa Clara, CA, USA), and sequencing was performed on the Illumina NovaSeq 6000 platform (Illumina Inc., San Diego, CA, USA). Raw reads were filtered with fastp to remove adapters and low-quality sequences. The resulting high-quality clean reads were utilized for subsequent analyses. (Shanghai Personalbio Technology Co., Ltd., Shanghai, China).

### 2.6. Quantitative Real-Time PCR (qRT-PCR)

Total RNA was extracted using TRIzol reagent and quantified with a NanoDrop 2000 (Thermo Fisher Scientific Inc., Waltham, MA, USA) spectrophotometer. cDNA was synthesized by reverse transcription. qRT-PCR was performed on a Roche LightCycler 480 (Roche Diagnostics GmbH, Mannheim, Germany) using SYBR Premix Ex Taq II (Takara Bio Inc., Kusatsu, Shiga, Japan) in 20 μL reactions containing primers, cDNA, and RNase-free water. The amplification program included an initial denaturation at 95 °C for 5 min, followed by 40 cycles of 95 °C for 15 s and 60 °C for 30 s. Melt curve analysis was performed to verify specificity of the amplification. The reference sequences used for the qRT-PCR are listed in Table S1. Three biological replicates were performed for each treatment, and data were normalized using ADPR as the internal reference gene [27]. Relative gene expression was determined by the 2^−ΔΔCt^ method.

### 2.7. Untargeted Metabolomics Profiling and Analysis

For metabolomic analysis, six pots per treatment (*n* = 6) were used as independent biological replicates. From each pot, roots from three plants were randomly selected and pooled to form a composite sample for each replicate. Root samples (80 mg) were thawed at 4 °C, homogenized in pre-chilled methanol/acetonitrile/water (2:2:1, *v*/*v*/*v*), sonicated for 30 min at low temperature, incubated at −20 °C for 10 min, and centrifuged at 14,000× *g* for 20 min at 4 °C. The supernatant was dried, reconstituted in 100 μL acetonitrile/water (1:1, *v*/*v*), vortexed, and centrifuged. UHPLC-Q-TOF MS analysis was performed using an Agilent 1290 (Agilent Technologies Inc., Santa Clara, CA, USA) Infinity system with a BEH C18 column (100 × 2.1 mm, 1.7 μm). The mobile phase consisted of a gradient from 5% to 100% methanol over 10 min, held for 2 min, then returned to initial conditions. The autosampler was maintained at 4 °C. Mass spectrometry was conducted in positive and negative electrospray ionization modes, scanning *m*/*z* 60–1000 with a spray voltage of ±5.5 kV. Quality control samples were regularly analyzed to ensure instrument stability (Figure S2). Data normalization was based on total peak intensity.

### 2.8. Combined Transcriptome and Metabolome Analysis

After performing quantitative analysis of the metabolomics and transcriptomics data, the initial step is to generate the quantitative results for both datasets, followed by correlation analysis and O2PLS analysis. The next step involves extracting information on differentially expressed metabolites and transcripts and filtering the correlation results. Subsequently, based on the metabolite information from the KEGG database, the corresponding enzyme-associated transcripts are retrieved. The differential expression trends of metabolites and transcripts with corresponding relationships were organized for further analysis. The shared pathways identified through differential enrichment analysis of both the transcriptome and metabolome were compiled, along with comprehensive pathway annotations for the differentially expressed metabolites and genes.

### 2.9. Statistical and Data Analysis

Statistical analyses of phenotypic, physiological, and qRT-PCR data were performed using GraphPad Prism 9. Differences among treatments were evaluated by one-way analysis of variance (ANOVA), and statistical significance was determined at *p* < 0.05. For transcriptomic analysis, differential gene expression was assessed using DESeq2 (version 1.38.3) based on FPKM values. Genes exhibiting |log2FoldChange| > 1 and *p*-value < 0.05 were considered differentially expressed [28]. Functional enrichment analyses, including GO and KEGG pathway analyses of DEGs, were conducted using the topGO (version 2.50.0) and clusterProfiler packages (version 4.6.0), with a significance threshold of *p* < 0.05. Data visualizations, such as Venn diagrams, heatmaps, and bar plots, were generated using the GenesCloud platform (www.genescloud.cn, accessed on 17 May 2024). For metabolomic profiling, multivariate analyses, including Pareto-scaled PCA and PLS-DA, were performed using the R package ropls (version 2.6.3). Differential metabolites were selected based on VIP > 1 and *p* < 0.05. Additional visualizations were created via the GenesCloud platform (www.genescloud.cn, accessed on 9 July 2024).

## 3. Results

### 3.1. The Role of AMF in Alleviating Saline–Alkali Stress and Promoting Oat Growth

**Figure 1:**
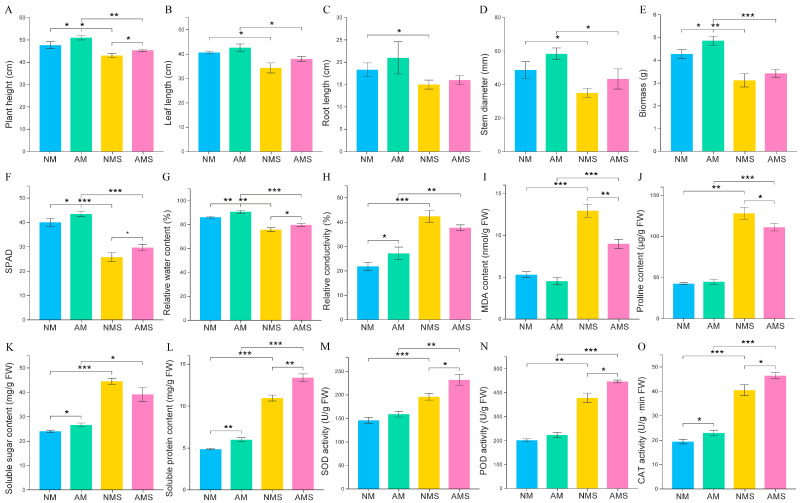
Comparative analysis of oat growth and physiological parameters under four different treatment conditions: non-mycorrhizal plants under normal conditions (NM), mycorrhizal plants under normal conditions (AM), non-mycorrhizal plants under saline–alkali stress (NMS), and mycorrhizal plants under saline–alkali stress (AMS). The measured parameters include oat plant height (**A**), leaf length (**B**), root length (**C**), stem diameter (**D**), biomass (**E**), SPAD value (**F**), leaf relative water content (**G**), leaf relative conductivity (**H**), root MDA content (**I**), root proline content (**J**), leaf soluble sugar (**K**) and protein contents (**L**), as well as root antioxidant enzyme activities, including SOD (**M**), POD (**N**), and CAT (**O**). (* *p* < 0.05, ** *p* < 0.01, *** *p* < 0.001).

To systematically investigate the effects of AMF in alleviating saline–alkali stress and promoting oat growth, we performed an integrated assessment of oat morphological characteristics, physiological responses, and antioxidant enzyme activities under different treatment conditions (Figure 1). Under both normal and saline–alkali stress conditions, oats inoculated with AMF exhibited higher plant height, leaf length, root length, stem diameter, and biomass compared to the non-inoculated control group (Figure 1A–E). Specifically, AM increased plant height and biomass by approximately 8.5% and 15.3%, respectively, compared to the NM group.

Additionally, AMF significantly increased SPAD values and relative water content of the leaves (Figure 1F,G), with significant differences observed in both NM vs. AM and NMS vs. AMS comparisons. Compared to the NMS group under saline–alkali stress, the AMS group showed increases of 16.7% in SPAD value and 7.3% in relative water content. Meanwhile, AM fungi colonization effectively reduced cell membrane damage markers, including relative conductivity and malondialdehyde (MDA) content (Figure 1H,I). Notably, MDA content in the AMS group was markedly reduced by approximately 35.8% compared to the NMS group.

Regarding osmotic regulators (Figure 1J–L), the levels of soluble sugar and soluble protein showed a slight increase under AM treatment, whereas proline content remained largely unchanged compared to the NM group. Under saline–alkali stress, proline content in the AMS group decreased by 13.3% compared to the NMS group, whereas soluble protein content increased by 21.8%. This indicates that AMF might enhance plant environmental adaptability by optimizing the composition of osmotic regulators. Analysis of the antioxidant enzyme system further revealed the protective mechanisms induced by AMF (Figure 1M–O). Under saline–alkali stress conditions, the AMS group exhibited increases of 18.4%, 18.2%, and 14.8% in SOD, POD, and CAT activities, respectively, relative to the NMS group. Notably, even under normal growth conditions, the AM group showed higher antioxidant enzyme activity than the NM group, indicating that AMF not only trigger defensive responses under stress but might also continuously enhance the plant’s basal antioxidant capacity.

### 3.2. Transcriptomic Analysis Under AMF Symbiosis

#### 3.2.1. Sequencing and Identification of Differentially Expressed Genes (DEGs)

**Figure 2:**
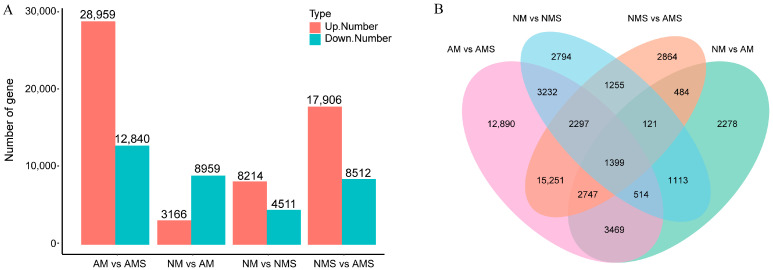
Transcriptome analysis under four different treatment conditions: non-mycorrhizal plants under normal conditions (NM), mycorrhizal plants under normal conditions (AM), non-mycorrhizal plants under saline–alkali stress (NMS), and mycorrhizal plants under saline–alkali stress (AMS). (**A**) Statistical summary of DEGs; (**B**) Venn diagram of DEGs among treatments.

To explore the differences between the AM, AMS, NM, and NMS treatment groups, RNA-seq sequencing analysis was performed (Figure 2). After quality control filtering, the average Q20 value was 97.60%, the average Q30 value was 93.61%, and the proportion of high-quality sequence bases (clean data) was 98.03% (Table S2). These results indicate that the obtained sequencing data is of high quality and provides a reliable foundation for subsequent analysis. Sample correlation analysis demonstrated high reproducibility within each treatment group, and differences existed between groups (Figure S3). Differential expression analysis between the NM and AM groups (Figure 2A) identified 8959 upregulated and 3166 downregulated genes, whereas in the NMS vs. AMS group, there were 17,906 upregulated DEGs and 8512 downregulated DEGs, indicating that AMF colonization induced a larger-scale gene expression change associated with the response to saline–alkali stress. Furthermore, the Venn diagram analysis (Figure 2B) showed that the NM vs. AM group and the NMS vs. AMS group had 2278 and 2864 unique DEGs, respectively, while 484 shared DEGs between the two groups might be associated with the common physiological regulatory mechanisms of AMF colonization and saline–alkali stress.

#### 3.2.2. KEGG and GO Pathway Enrichment Analysis of Differentially Expressed Genes (DEGs)

**Figure 3:**
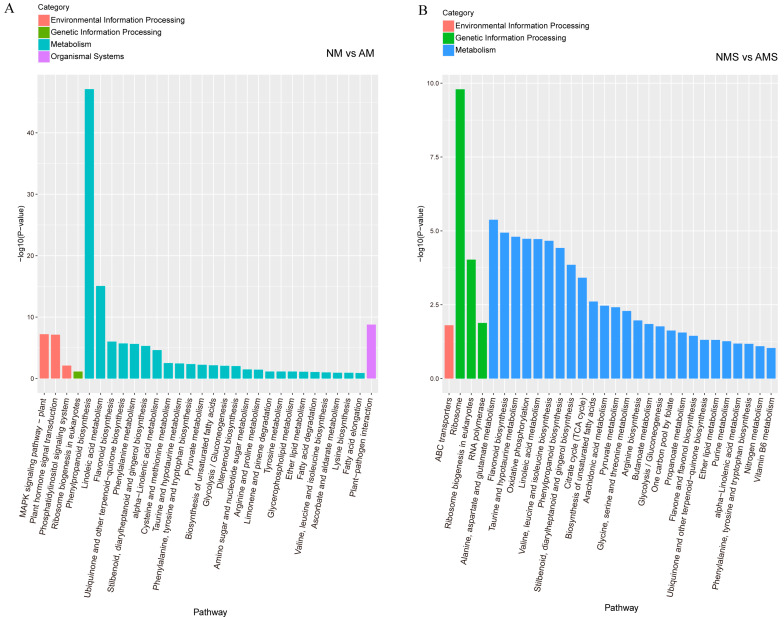
Pathway enrichment analysis under different treatments: non-mycorrhizal plants under normal conditions (NM), mycorrhizal plants under normal conditions (AM), non-mycorrhizal plants under saline–alkali stress (NMS), and mycorrhizal plants under saline–alkali stress (AMS). Panels (**A**,**B**) represent the KEGG pathway enrichment analysis for NM vs. AM and NMS vs. AMS, respectively.

KEGG pathway enrichment analysis was conducted to functionally categorize DEGs in the NM vs. AM and NMS vs. AMS comparison groups (Figure 3A,B). In the NM vs. AM comparison (Figure 3A), pathways classified under the metabolism category were the most enriched. Notably, phenylpropanoid biosynthesis and linoleic acid metabolism exhibited significant enrichment. Additionally, pathways involved in Environmental Information Processing, such as the MAPK signaling pathway, were also prominently enriched. In contrast, only one pathway was annotated in each category under the Genetic Information Processing and Organismal Systems categories.

In the NMS vs. AMS analysis (Figure 3B), the metabolism category remained dominant, with pathways such as alanine, aspartate and glutamate metabolism, and flavonoid biosynthesis significantly enriched. Additionally, the ribosome pathway in the Genetic Information Processing category showed the highest significance, while only ABC transporters were annotated in the Environmental Information Processing category. Overall, these findings suggest that metabolic reprogramming is a prominent feature in both comparisons, while significant differences were observed between groups in terms of signaling and Genetic Information Processing pathways.

**Figure 4:**
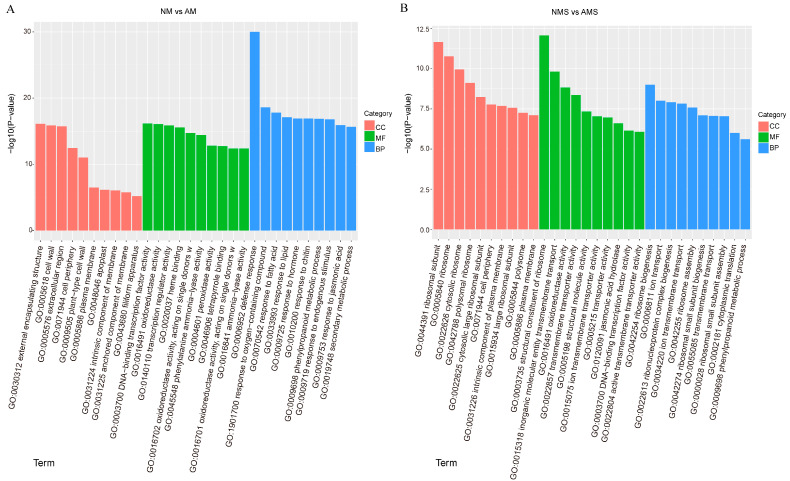
Pathway enrichment analysis under different treatments: non-mycorrhizal plants under normal conditions (NM), mycorrhizal plants under normal conditions (AM), non-mycorrhizal plants under saline–alkali stress (NMS), and mycorrhizal plants under saline–alkali stress (AMS). Panels (**A**,**B**) represent the GO Term enrichment analysis for NM vs. AM and NMS vs. AMS, respectively.

GO enrichment analysis was conducted for DEGs in both NM vs. AM and NMS vs. AMS comparisons (Figure 4A,B). In the NM vs. AM comparison (Figure 4A), the DEGs were significantly enriched in cellular components such as the cell wall, external encapsulating structure, and extracellular region. For molecular functions (MF), the DEGs were mainly associated with oxidoreductase activity, transcription regulator activity, DNA-binding transcription factor activity, and heme binding. The most enriched biological processes (BP) were response to oxygen-containing compound, phenylpropanoid metabolic process, and defense response, with defense response showing the highest significance.

In contrast (Figure 4B), the DEGs in the NMS vs. AMS group were mainly enriched in ribosomal subunit, cytosolic ribosome, and large ribosomal subunit, all associated with ribosomal cellular components. In terms of molecular function, the structural constituent of the ribosome was the most significantly enriched pathway, while other pathways, such as inorganic molecular entity transport and oxidoreductase activity, were also annotated. For biological processes, ribosome biogenesis showed the highest level of significance, with additional involvement of functions like transmembrane transport and oxidoreductase activity. These GO enrichment patterns highlight the distinct physiological responses between treatment groups. While the NM vs. AM comparison emphasizes defense and metabolic adaptation, the NMS vs. AMS group shows a strong transcriptional investment in ribosome biogenesis and protein synthesis.

#### 3.2.3. Transcription Factor Family Analysis and qRT-PCR Validation

**Figure 5:**
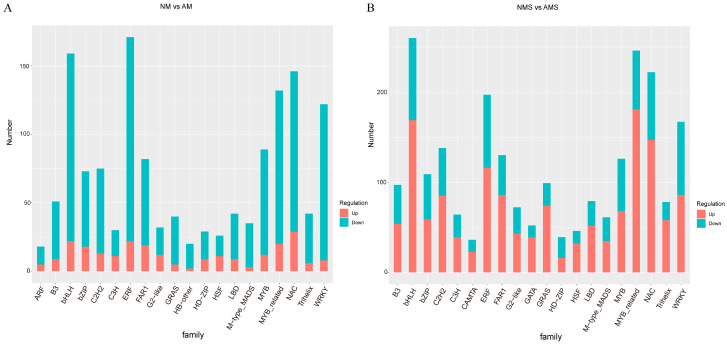
Panels (**A**,**B**) show the transcription factor families in NM vs. AM and NMS vs. AMS, respectively. NM: non-mycorrhizal plants under normal conditions; AM: mycorrhizal plants under normal conditions; NMS: non-mycorrhizal plants under saline–alkali stress; AMS: mycorrhizal plants under saline–alkali stress.

The regulatory patterns of different transcription factor families among DEGs under the two treatment conditions displayed significant differences (Figure 5A,B). In the NM vs. AM comparison (Figure 5A), such as bHLH, NAC, WRKY, ERF, and MYB, were predominantly downregulated. Conversely, in the NMS vs. AMS treatment (Figure 5B), these same families were mainly upregulated, and the overall number of annotated DEGs was higher than in the NM vs. AM comparison. These findings suggest that under saline–alkali stress, mycorrhizal symbiosis may enhance plant adaptability by promoting the upregulation of transcription factors, further activating specific metabolic or defense-related pathways.

For validation of transcriptome sequencing reliability, 10 DEGs were randomly selected based on gene expression patterns, and expression analysis was performed using qRT-PCR (Figure S4). Although there were some differences in the specific values and variation ranges between the FPKM values and the relative expression levels from qRT-PCR under different treatments, the overall expression trends were consistent and showed high correlation. These results confirm the accuracy and robustness of the transcriptomic data.

### 3.3. Metabolomic Analysis Under AMF Symbiosis

#### 3.3.1. Sequencing and Identification of Metabolites

**Figure 6:**
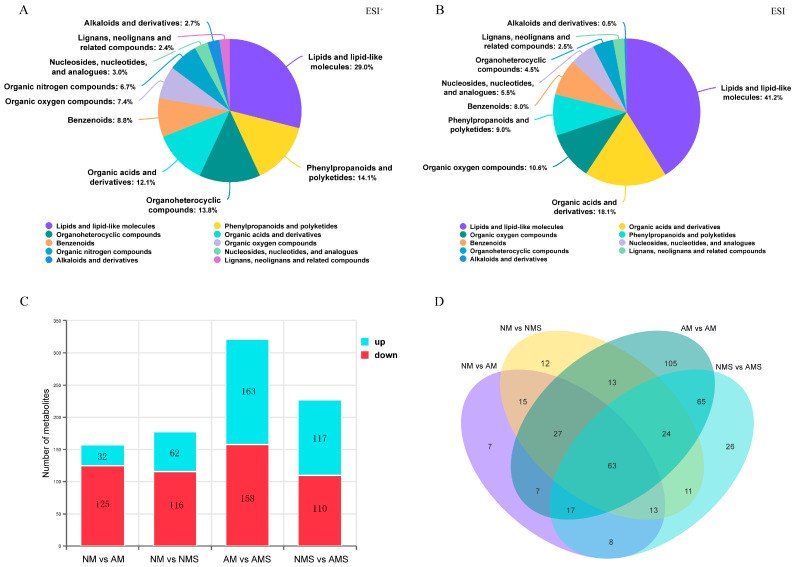
Metabolomics analysis under different treatment conditions: non-mycorrhizal plants under normal conditions (NM), mycorrhizal plants under normal conditions (AM), non-mycorrhizal plants under saline–alkali stress (NMS), and mycorrhizal plants under saline–alkali stress (AMS). The chemical classification distribution of metabolites in the ESI^+^ (**A**) and ESI^−^ (**B**) modes is shown. Panel (**C**) shows the statistical analysis of the number of DEMs between groups, and panel (**D**) presents the Venn diagram of DEMs between groups.

High-resolution non-targeted metabolomics was used to analyze the root system of oats to identify metabolites associated with growth promotion and saline–alkali stress, aiming to elucidate the mechanisms of AMF-mediated root adaptation (Figure 6). Each treatment group (NM, AM, NMS, AMS) was replicated six times to ensure statistical reliability. A total of 573 metabolites were annotated from the combined positive and negative ion mode analyses (Table S3). These metabolites primarily included lipids and lipid-like molecules, organic acids and derivatives, organheterocyclic compounds, phenylpropanoids and polyketides, nucleosides, nucleotides, and analogs (Figure 6A,B). The types of components in the two modes were similar, but the proportions showed significant differences. 3D-PCA analysis revealed clear separation among the four treatment groups under both positive and negative ion detection (Figure S5A,B), indicating substantial metabolic differences among them.

Further PLS-DA permutation testing (Figure S6) showed a gradual decline in R2 and Q2 for the random model, confirming the model’s robustness. The focus was placed on the number of differential metabolites (DEMs) in the NM vs. AM and NMS vs. AMS groups (Figure 6C). In the NM vs. AM treatment, 157 DEMs were annotated, with 32 metabolites upregulated and 125 downregulated, indicating a significant trend of metabolic downregulation between the NM and AM groups. In the NMS vs. AMS comparison, the number of up- and downregulated metabolites between the two groups was relatively balanced, with 117 upregulated and 110 downregulated, totaling 227 metabolites. Further analysis of the number of DEMs between groups (Figure 6D) showed 63 unique DEMs monitored across all groups. The NM vs. AM group had 15 unique DEMs, while the NMS vs. AMS group had 26 unique DEMs, indicating that under saline–alkali stress, AMF colonization led to the regulation of more metabolites in the oat root system.

#### 3.3.2. Analysis of the Types of Differential Metabolites (DEMs)

**Figure 7:**
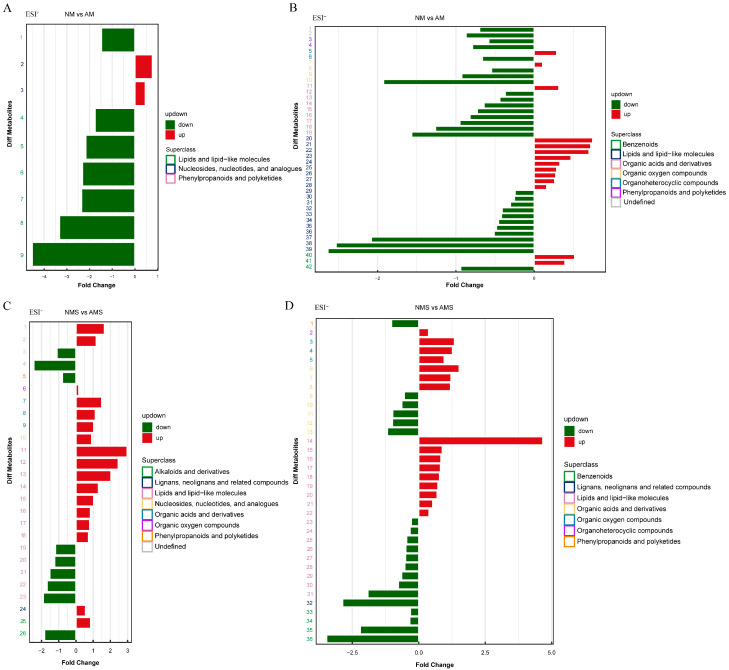
Classification of DEMs under different treatments: non-mycorrhizal plants under normal conditions (NM), mycorrhizal plants under normal conditions (AM), non-mycorrhizal plants under saline–alkali stress (NMS), and mycorrhizal plants under saline–alkali stress (AMS). Panels (**A**,**B**) represent the classification of DEMs between NM vs. AM in the ESI^+^ and ESI^−^ modes, respectively. Panels (**C**,**D**) represent the classification of DEMs between NMS vs. AMS in the ESI^+^ and ESI^−^ modes, respectively. Upregulated expression is shown in red, and downregulated expression is shown in green.

Further analysis of DEMs between NM vs. AM sand NMS vs. AMS groups was performed (Figure 7, Table S4). Significant expression differences in various metabolite categories were observed between the NM vs. AM and NMS vs. AMS groups in both the positive and negative ion modes of the metabolomics analysis. In the NM vs. AM comparison, the positive ion mode (Figure 7A) showed downregulation of metabolites in the lipids and lipid-like molecules and phenylpropanoids and polyketides categories, while a few metabolites in the Nucleosides, nucleotides, and analogs category were upregulated. In the negative ion mode (Figure 7B), the upregulation and downregulation trends of different metabolite categories were more pronounced. Specifically, 11 metabolites in the lipids and lipid-like molecules category were downregulated, and 9 were upregulated, while metabolites in the phenylpropanoids and polyketides, Organic acids and derivatives, and most organic oxygen compounds categories were downregulated.

In the NMS vs. AMS comparison, positive ion mode (Figure 7C) showed upregulation of the organic acids and derivatives category, while metabolites in the lipids and lipid-like molecules and alkaloids and derivatives categories displayed both up- and downregulation. In the negative ion mode (Figure 7D), the metabolites exhibited a more scattered up- and downregulation pattern, with the most annotated category still being lipids and lipid-like molecules. In the organic oxygen compounds category, metabolites were primarily upregulated, while Benzenoids and some organic acids and derivatives metabolites were mostly downregulated. Overall, significant metabolic differences were observed between the NM vs. AM and NMS vs. AMS groups, especially in the lipid, organic compound, and phenolic metabolites, further revealing the physiological functional differences induced by AMF under different treatments.

#### 3.3.3. KEGG Enrichment Analysis of Differential Metabolites (DAMs)

**Figure 8:**
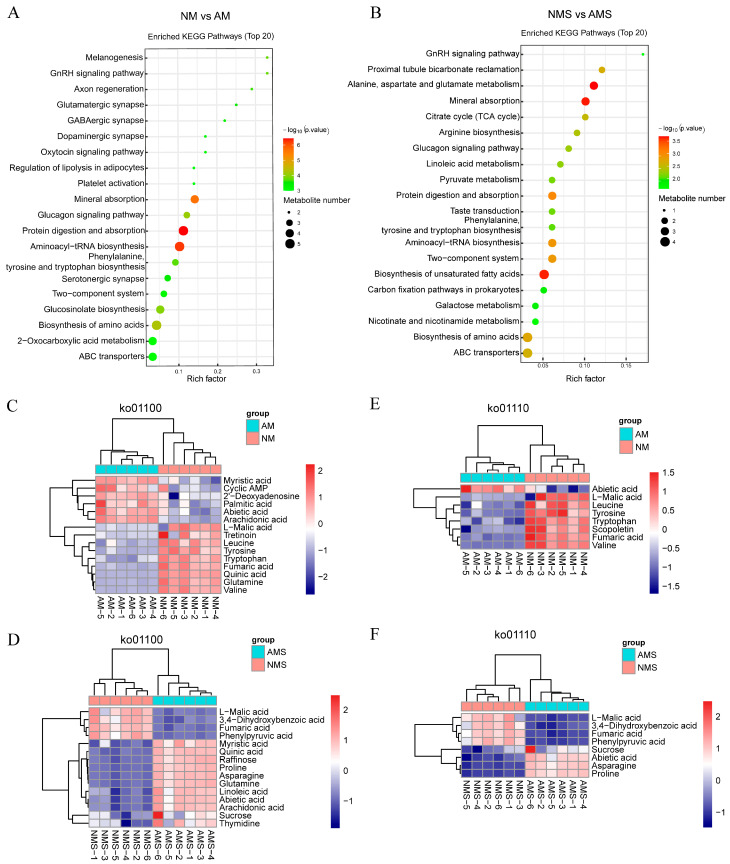
Analysis of KEGG enrichment pathways and differences in metabolite content under different treatment conditions: non-mycorrhizal plants under normal conditions (NM), mycorrhizal plants under normal conditions (AM), non-mycorrhizal plants under saline–alkali stress (NMS), and mycorrhizal plants under saline–alkali stress (AMS). Panels (**A**,**B**) show the KEGG pathway enrichment analysis for NM vs. AM and NMS vs. AMS, respectively. The heatmaps display the relative content of DEMs in specific metabolic pathways (ko01100, (**C**,**D**); ko01110, (**E**,**F**)).

KEGG enrichment analysis of DAMs in NM vs. AM and NMS vs. AMS comparisons revealed distinct pathway-level metabolic reprogramming (Figure 8A,B). In NM vs. AM (Figure 8A), the pathways Protein digestion and absorption and Aminoacyl-tRNA biosynthesis were significantly enriched with metabolites and had lower *p*-values, indicating that these pathways exhibited significant differences between the groups. Additionally, pathways such as biosynthesis of amino acids, mineral absorption, 2-oxocarboxylic acid metabolism, and ABC transporters also showed significant enrichment, suggesting differences between the two groups in terms of energy metabolism, mineral utilization, and transmembrane transport. In NMS vs. AMS (Figure 8B), pathways such as mineral absorption and alanine, aspartate, and glutamate metabolism pathways were significantly enriched. Notably, the biosynthesis of unsaturated fatty acids pathway exhibited the greatest number and enrichment of metabolites, suggesting its critical role in modulating lipid metabolism and membrane fluidity under stress. Furthermore, biosynthesis of amino acids and ABC transporter pathways also showed considerable enrichment.

Focusing on KEGG metabolic pathways with more than five DEMs (Figure 8C–F, Table S5), the expression level differences in significant metabolites in “metabolic pathways” (ID: ko01100) and “biosynthesis of secondary metabolites” (ID: ko01110) were analyzed for both treatment groups. In the differential metabolite clustering heatmap for the AM vs. NM group (Figure 8C), significant expression level differences in metabolites between the two groups were observed. Myristic acid, cyclic AMP, 2′−deoxyadenosine, palmitic acid, and arachidonic acid were highly expressed in the AM group, indicating more active lipid metabolism, signal transduction, and DNA metabolism in the AM group compared to the NM group. In the heatmap for the AMS vs. NMS group (Figure 8D), metabolites like myristic acid, linoleic acid, and proline were more highly expressed in the AMS treatment, reflecting significant physiological differences in lipid metabolism, cell membrane stability, and stress responses between the AMS and NMS treatments. Moreover, the pathway analysis for the “biosynthesis of secondary metabolites” showed that abietic acid expression was relatively high in both treatment groups (Figure 8E,F). In the heatmap for the “AMS vs. NMS” group (Figure 8F), asparagine and proline also showed higher expression levels, which may reflect the importance of these metabolites in adaptive metabolism and stress responses.

### 3.4. Integrated Analysis of Transcriptome and Metabolome

The DEGs and DAMs were analyzed through co-expression network analysis. In both NM vs. AM and NMS vs. AMS, DEGs and DAMs in quadrants three and seven displayed consistent trends, suggesting positive regulation of metabolite accumulation by gene expression (Figure S7). A subsequent KEGG enrichment analysis of these correlated gene-metabolite pairs (Figure S8) revealed that in the NM vs. AM treatment, the phenylpropanoid biosynthesis pathway was significantly enriched. In contrast, in the NMS vs. AMS group, metabolic pathways such as alanine, aspartate and glutamate metabolism, valine, leucine and isoleucine biosynthesis, the citrate cycle, and pyruvate metabolism were annotated with a relatively large number of DEGs and DAMs.

**Figure 9:**
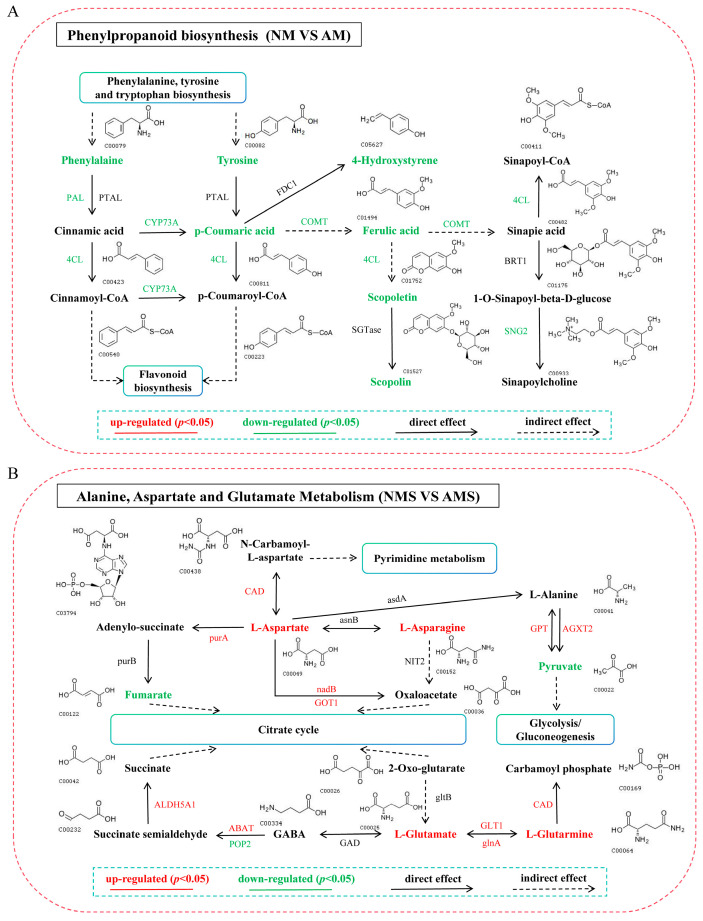
Correlation analysis of DEGs and DEMs. (**A**) Analysis of the phenylpropanoid biosynthesis pathway under NM vs. AM treatment, (**B**) Analysis of the alanine, aspartate, and glutamate metabolism pathway under NMS vs. AMS treatment. Red font indicates upregulation, green font indicates downregulation, solid lines represent direct effects, and dashed lines represent indirect effects. NM: non-mycorrhizal plants under normal conditions; AM: mycorrhizal plants under normal conditions; NMS: non-mycorrhizal plants under saline–alkali stress; AMS: mycorrhizal plants under saline–alkali stress.

Based on this, a detailed analysis of the phenylpropanoid biosynthesis pathway (NM vs. AM) (Figure 9A) showed downregulation of key enzyme-encoding genes such as PAL, 4CL, CYP73A, and COMT, leading to decreased levels of related metabolites including phenylalanine, tyrosine, p-coumaric acid, 4-hydroxystyrene, ferulic acid, and scopolin, indicating overall inhibition of the pathway. On the other hand, in the NMS vs. AMS treatment (Figure 9B), key genes in the alanine, aspartate and glutamate metabolism pathway, including CAD, purA, nadB, ABAT, and POP2, were regulated to varying degrees. Metabolites such as L-asparagine, L-aspartate, L-glutamine and L-glutamate were significantly upregulated, while fumarate and pyruvate were downregulated. These results suggest that AM may enhance the plant stress tolerance by promoting the biosynthesis and accumulation of specific amino acids under saline–alkali stress.

## 4. Discussion

This study demonstrates that arbuscular mycorrhizal fungi significantly alleviate the detrimental effects of saline–alkali stress on oat (*Avena sativa* L.) growth through a multifaceted regulatory network, specifically in the aspects of morphological development, osmotic regulation, cell membrane stability, and antioxidant systems. Under both normal and stress conditions, AMF inoculation markedly improved oat plant height, root length, and aboveground biomass. This effect is likely due to their symbiotic structure (mycelial network) that facilitates the efficient transport of water and mineral nutrients, particularly phosphorus [29]. Under saline–alkali stress, phosphorus is often in an unavailable state [30], but AM symbiosis can significantly improve its availability, thereby supporting the continuous process of photosynthesis and carbon assimilation, which sustains growth [16,31]. The significant increase in SPAD value and leaf RWC in this study further suggests that AMF play an important role in maintaining the stability of the photosynthetic system and water potential balance, potentially through the regulation of aquaporin expression, which enhances the root’s water absorption capacity [32].

Osmotic adjustment was identified as a primary adaptive strategy utilized by plants to mitigate the effects of saline–alkali stress [30,33]. In this study, AM inoculation generally increased levels of proline, soluble sugars, and soluble proteins. Notably, proline levels were lower in the AMS group compared to the NMS group, indicating that AMF may modulate osmolyte composition and allocation to improve stress adaptation. Although the accumulation of high levels of proline can alleviate osmotic stress, it also imposes a significant burden on energy metabolism. This “energy-saving” adaptation mechanism may be achieved by regulating key enzymes such as pyrroline-5-carboxylate synthetase (P5CS) and proline dehydrogenase (ProDH) [34,35,36].

Regarding membrane stability, saline–alkali stress often induces excessive accumulation of ROS, leading to lipid peroxidation and membrane damage. To cope with oxidative stress induced by oxygen-containing compounds, cellular receptors or redox-sensitive proteins recognize elevated ROS levels and activate associated signaling pathways. These pathways trigger various transcription factors that regulate antioxidant gene expression. Consequently, cells enhance their ROS scavenging capacity by upregulating the synthesis and activity of enzymatic antioxidants such as SOD, CAT, and POD, while also increasing levels of non-enzymatic antioxidants to directly neutralize excess ROS. Additionally, metabolic pathways are adjusted to boost the production of reducing molecules like NADPH, ensuring the sustained operation of antioxidant systems. When necessary, repair mechanisms are initiated to restore oxidatively damaged biomolecules, or programmed cell death is activated to eliminate severely damaged cells, thereby maintaining overall tissue homeostasis [37,38].

In this study, AM inoculation significantly enhanced the activities of SOD, POD, and CAT, indicating that AMF play a central role in regulating redox balance. Previous studies have shown that AMF symbiosis can activate the expression of genes related to antioxidant defense, thereby making plants more proactive and adaptive under saline–alkali conditions [39,40]. Notably, the activation of the antioxidant system by AMF was not only evident under stress conditions but also exhibited higher enzyme activities in the inoculated group under normal conditions. This phenomenon suggests that AMF symbiosis may confer “pre-adaptive” capacity by continuously activating basal defense systems, enabling plants to respond more rapidly and efficiently when encountering stress. This mechanism is likely associated with systemic acquired tolerance (SAT), highlighting the pivotal role of AMF in inducible plant defense [41]. Meanwhile, in future studies, the application of exogenous antioxidants (such as N-acetylcysteine) may also help elucidate the role of ROS scavenging in stress tolerance and reveal potential interactions with AMF symbiosis.

Differential expression analysis revealed that the NMS vs. AMS comparison identified more DEGs than the NM vs. AM comparison, suggesting that AMF induced more extensive transcriptional reprogramming under saline–alkali stress. KEGG analysis revealed that AM symbiosis mainly influenced metabolic pathways, along with notable changes in signal transduction and genetic information processing. In the NM vs. AM group, the enrichment of linoleic acid metabolism and the MAPK signaling pathway suggests that AMF may regulate early stress perception and response through oxylipin signaling and MAPK cascade amplification [42]. In contrast, in the NMS vs. AMS group, the ribosome pathway was significantly enriched in AMS plants, indicating that under saline–alkali stress, AMF may promote ribosome biogenesis and enhance translational activity, thereby supporting the production of stress-responsive proteins [43].

GO analysis further revealed the broad regulation of gene expression by AMF across the three major categories. In the NM vs. AM comparison, enrichment in terms such as cell wall and external encapsulating structure suggests that AMF may enhance the external barrier function of plants by regulating the expression of cell wall-associated proteins [44]. Additionally, the enrichment of oxidoreductase activity and response to oxygen-containing compounds highlights the role of AMF in modulating ROS homeostasis [45]. In the NMS vs. AMS comparison, GO terms related to ribosome structure and function, such as ribosomal subunit and structural constituent of ribosome, were significantly enriched, further emphasizing the central role of translation in AMF-mediated stress alleviation. Moreover, enrichment in transmembrane transport and oxidoreductase activity suggests that AMF may regulate membrane transport systems, thereby improving intracellular homeostasis and metabolic adjustments under stress conditions [39,46].

AMF colonization also reprogrammed the metabolic networks of oat roots under both normal and saline–alkali stress conditions. The number of differentially abundant metabolites increased, indicating a broader and more intense regulation of host metabolism under adverse conditions. Under both conditions, AMF significantly influenced lipid metabolism, signaling molecules, and amino acid profiles. Notably, “lipids and lipid-like molecules” were generally downregulated under non-stress conditions, suggesting that AMF may suppress lipid peroxidation and phenolic metabolism to reduce oxidative load, thereby maintaining energy homeostasis [47]. In contrast, under saline–alkali stress, lipid metabolism showed a complex pattern of both up- and downregulation. Several polyunsaturated fatty acids, such as myristic acid and linoleic acid, were significantly upregulated, potentially enhancing membrane flexibility and stability [48].

Changes in signaling molecules and nucleoside metabolites further revealed the importance of AMF in regulating rhizosphere signaling and cellular metabolism. The significant upregulation of cyclic AMP and 2′-deoxyadenosine in AM-inoculated plants may contribute to the maintenance of symbiotic structures. As a classical second messenger, elevated cAMP levels are known to regulate plasma membrane cyclic nucleotide-gated channels (CNGCs) and activate downstream protein kinase A (PKA) signaling cascades. This regulation modulates various cellular metabolism pathways, including ion transport, calcium signaling, and ROS homeostasis, thereby adjusting metabolic activities to meet the physiological demands under symbiotic conditions [49]. Furthermore, cAMP-mediated signaling influences the expression of genes involved in energy metabolism and stress responses, promoting an adaptive metabolic state favorable for both plant and fungal partners [50]. Regarding root signaling, cAMP plays key roles by modulating ion channel activity, which controls nutrient uptake and root growth dynamics. The regulation of CNGCs by cAMP affects cytosolic calcium levels, a pivotal messenger in root development and environmental response signaling pathways. This enables the plant to sense and respond to changes in soil nutrient availability and abiotic stresses efficiently. Additionally, cAMP-PKA signaling influences root architecture by regulating cell division and elongation processes, thus optimizing root system development to enhance water and nutrient acquisition in the rhizosphere [49]. Simultaneously, the upregulation of 2′-deoxyadenosine indicates enhanced nucleic acid metabolism, supplying substrates for DNA replication and repair to sustain active cell division and growth during root development and symbiosis [51].

Combined analyses further revealed that in the NM vs. AM comparison, the expression of key enzyme-coding genes and the accumulation of downstream metabolites in the phenylpropanoid pathway were generally downregulated, suggesting that phenylpropanoid metabolism was suppressed under AM symbiosis. This result further suggests that host regulation mediated by AMF shows more coordinated and resource-efficient characteristics, optimizing resource allocation to maintain essential metabolism.

In the NMS vs. AMS comparison, pathways related to amino acid metabolism were significantly enriched, particularly the alanine, aspartate, and glutamate metabolism pathway. Previous research has demonstrated that glutamate and its derived amino acids play central roles in plant stress responses, serving not only as hubs for nitrogen metabolism to support nitrogen allocation and recycling but also as precursors for antioxidants such as glutathione, thus enhancing cellular ROS scavenging capacity. In particular, glutathione (GSH), a tripeptide antioxidant, achieves this either through direct non-enzymatic reduction in ROS or by serving as a cofactor for glutathione peroxidase (GPX), which converts peroxides to water or alcohols while oxidizing GSH to glutathione disulfide (GSSG). Subsequently, glutathione reductase (GR) recycles GSH from GSSG, thus sustaining the antioxidant system [52,53].

Aspartate has also been identified as a key precursor for the biosynthesis of several essential amino acids (e.g., lysine, threonine, and methionine) and asparagine, providing carbon skeletons and energy support for plants [54]. Notably, key intermediates of the TCA cycle, such as fumarate and pyruvate, showed a decreasing trend. Pyruvate, the end product of glycolysis and an important entry point into the TCA cycle, and fumarate, a critical intermediate within the TCA cycle. The observed decrease in their abundance may reflect a strategy by the host plant under AMF symbiosis to actively adjust carbon flow, reducing the accumulation of certain TCA cycle intermediates and allocating more resources toward the synthesis of protective compounds such as amino acids [55,56]. Moreover, previous studies have indicated that AMF can enhance plant adaptation to various stresses by regulating the accumulation of small molecules like amino acids and sugars [16]. The findings of this study further support this notion. Meanwhile, future studies could consider the exogenous application or inhibition of key metabolites to further validate their direct roles in oat stress adaptation under AMF symbiosis.

At a deeper level, AMF may further optimize root adaptation to saline–alkali stress by modulating the coupling between endogenous hormone signaling and transcriptional networks. Previous studies have reported that AMF colonization can trigger changes in host hormone levels, including ABA, IAA, and GA, thereby coordinating stomatal movement, cell division, and defense responses [57]. In this study, the significant accumulation of organic acids and derivatives in the AMS group suggests that these metabolites may not only participate in chelating soil mineral elements but also serve as molecular scaffolds for the biosynthesis of hormone precursors [58,59,60]. To validate the coupling effects between metabolism and gene expression, future research could dynamically monitor the expression of key rate-limiting enzyme genes using molecular techniques, aiming to construct an integrated network of metabolism and transcriptional regulation.

Additionally, to quantitatively assess AMF-mediated metabolic reallocation, ^13C and ^15N stable isotope tracing and metabolic flux analysis (MFA) could be employed to precisely track the dynamic distribution of carbon and nitrogen among lipid synthesis, amino acid metabolism, and secondary metabolite production [61,62]. This approach would not only validate the findings of the untargeted metabolomic analyses at the molecular level but also provide quantitative evidence and theoretical support for the efficient application of AMF in improving crop resilience in saline–alkali soils.

## 5. Conclusions

This study systematically revealed the multidimensional mechanisms by which AMF alleviate saline–alkali stress and promote oat growth. At the physiological level, AMF symbiosis significantly enhanced water use efficiency, osmotic regulation capacity, membrane stability, and antioxidant defense in oats, providing critical support for maintaining growth under saline–alkali conditions. Transcriptomic and metabolomic analyses further demonstrated that AMF exerted “condition-dependent” regulation. Under normal conditions, AMF primarily optimized basal metabolism and resource allocation to sustain plant growth. In contrast, under saline–alkali stress, AMF enhanced amino acid metabolism and the accumulation of signaling molecules, improved regulation of the antioxidant defense system and transmembrane transport, and strengthened the transcriptional network of stress-responsive transcription factors. These changes collectively reconstructed the host’s stress-responsive transcriptional and metabolic systems. Collectively, AMF confer enhanced growth capacity and saline–alkali tolerance to oats through core strategies of energy optimization, signal reprogramming, and metabolic remodeling. This study deepens our understanding of the mechanisms underlying AMF–plant interactions under saline–alkali stress and provides new insights into how AMF may enhance crop tolerance and productivity in the future.

## Data Availability

The original contributions presented in this study are included in the article/Supplementary Material. Further inquiries can be directed to the corresponding author.

## Supplementary Materials

The following supporting information can be downloaded at https://www.mdpi.com/article/10.3390/jof11080587/s1, Table S1: Primers used in the qRT-PCR assay. Table S2: Quality summary of transcriptome sequencing data. Table S3: Statistics of the number of metabolites identified in positive and negative ion modes. Table S4: Annotation of differential metabolites between different treatments under positive and negative ion modes (corresponding to Figure 7). Table S5: Statistical analysis of metabolite quantification under different treatments (corresponding to Figure 8). Figure S1: (A) Plant phenotypes under different treatments; (B) representative micrographs of samples stained with trypan blue; (C) AMF colonization rate under different treatments. (* *p* < 0.05, ** *p* < 0.01, *** *p* < 0.001). Figure S2: Quality control (QC) sample correlation analysis in ESI^+^ (A) and ESI^−^ (B) modes. Figure S3: Analysis of correlations among different treatment groups. Figure S4: (A–J) Transcription levels of 10 DEGs were analyzed by qRT-PCR and RNA-seq (* *p* < 0.05, ** *p* < 0.01, *** *p* < 0.001). Figure S5: The 3D-PCA distribution of different treatment groups (AM, AMS, NM, NMS) and quality control samples (QC) in the ESI^+^ (C) and ESI^−^ (D) modes. Figure S6: PLS-DA permutation tests under different ionization modes. (A–B): NM vs. AM; (C–D): NMS vs. AMS (positive and negative ion modes, respectively). Figure S7: Nine-quadrant analysis plots. (A) NM vs. AM; (B) NMS vs. AMS. Figure S8: KEGG-based correlation analysis of DEGs and DAMs, (A) NM vs. AM; (B) NMS vs. AMS.
