# Supplementary material for: Transcriptomic and Metabolomic Insights into the Effects of Arbuscular Mycorrhizal Fungi on Root Vegetative Growth and Saline–Alkali Stress Response in Oat (Avena sativa L.)
Source: J Fungi (Basel). 2025 Aug 9;11(8):587. doi: 10.3390/jof11080587 (PMC12387890; doi:10.3390/jof11080587)
Supplement: Supplementary file 1 [file jof-11-00587-s001.zip › SUPPLEMENTARYINFORMATION.pdf]

SUPPLEMENTARY INFORMATION

TABLES

TABLE S1 Primers used in the qRT-PCR assay.

ID	Gene ID	Primer Sequences (5′ to 3′)
1	DN691_c4_g1	F：GAGAAAGAACCCGAGGAC<br>R：GGGACAGTGAAAGCAGTA
2	DN257_c0_g1	F：TCCCTATGAATCTGAAAG<br>R：TACATTATGGTCTTGGAA
3	DN1195_c1_g1	F：CCAAGAGGCAGATAAGGCTAA<br>R：AACATACACGCACATACTACAAC
4	DN658_c2_g2	F：ATGTTGGGGTAGAAGAGC<br>R：ATCGAGACCTACGTGTTC
5	DN38445_c0_g1	F：GGTGATGAAGGAGAAGGT<br>R：GCTTATTCGCCTCACCTT
6	DN10626_c0_g1	F：CGTCAAAGCAACAACTCCAGAA<br>R：AAGCACCGGAGCAGAGAC
7	DN1764_c1_g2	F：CTCTGCCACCTCAAGTTC<br>R：AGGAAGAGAGCGATGAAGA
8	DN498_c2_g1	F：AGGATATTACTCTCAGGATTA<br>R：GGTTACGCTTGATAATGT
9	DN2511_c0_g1	F：CGAAGAAGAAGGACAAGGGC<br>R：CGCTCGAATTCACCTCCATG
10	DN19121_c0_g1	F：GCAACGTCTTCCAGCAGG<br>R：GTTCATGACGGCGTCCTTG
11	ADPR	F：CTCATGGTTGGTCTCGATGC<br>R：ACATCCCAAACAGTGAAGCT

TABLE S2 Quality Summary of transcriptome sequencing data.

Sample	Reads No.	Clean Reads No.	Clean Reads %	Clean Data %	N (%)	Q20 (%)	Q30 (%)
NM_1	49477226	48659264	98.35	98.12	0.000753	97.85	94.14
NM_2	50660198	49871524	98.44	98.23	0.000823	97.79	93.88
NM_3	62466862	61343294	98.20	98.00	0.000786	97.62	93.540
AM_1	47080064	46220674	98.17	97.96	0.000766	97.55	93.35
AM_2	45614874	44848260	98.32	98.12	0.000772	97.76	93.88
AM_3	45389094	44518436	98.08	97.88	0.000792	97.39	92.97
NMS_1	52955210	52019460	98.23	97.98	0.000793	97.70	93.76
NMS_2	59719992	58705046	98.30	98.04	0.000760	97.70	93.73
NMS_3	48829760	48000714	98.30	98.06	0.000779	97.79	93.99
AMS_1	43661110	42957374	98.39	98.17	0.000772	97.78	93.89
AMS_2	53582758	52545126	98.06	97.84	0.000801	97.45	93.16
AMS_3	49265310	48336089	98.11	97.89	0.000812	97.42	93.04

TABLE S3 Statistics of the Number of Metabolites Identified in Positive and Negative Ion Modes

Detection mode	Metabolite count
Positive ion mode （ESI^+）	333
Negative ion mode （ESI^-）	240

TABLE S4 Annotation of differential metabolites between different treatments under positive and negative ion modes(corresponding to Figure 7).

TABLE S5 Statistical analysis of metabolite quantification under different treatments(corresponding to Figure 8).

FIGURE

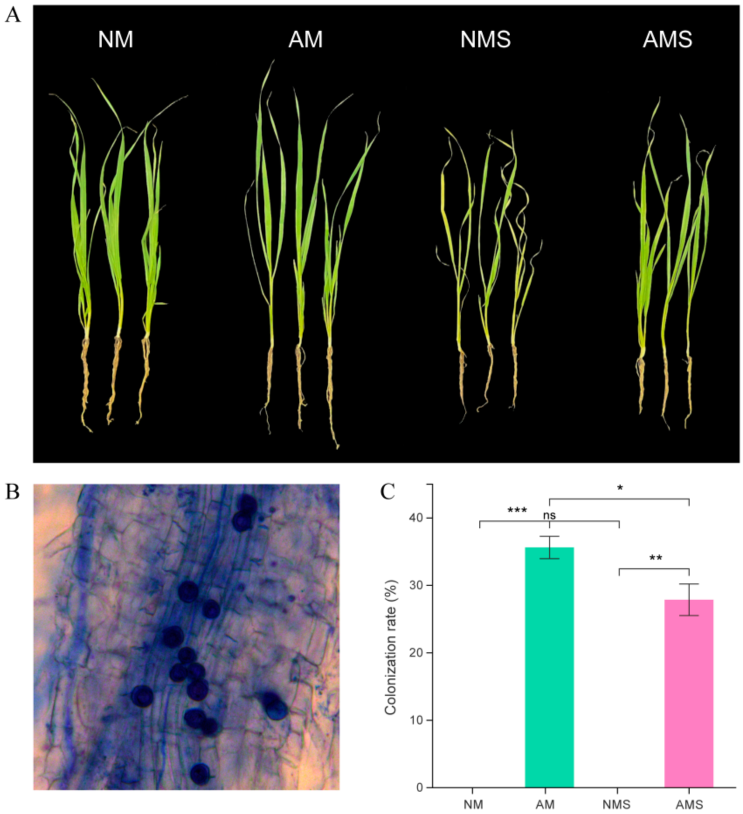

FIGURE S1 (A) Plant phenotypes under different treatments; (B) representative micrographs of samples stained with trypan blue; (C) AMF colonization rate under different treatments. (*$p < 0.05$, **$p < 0.01$, ***$p < 0.001$).

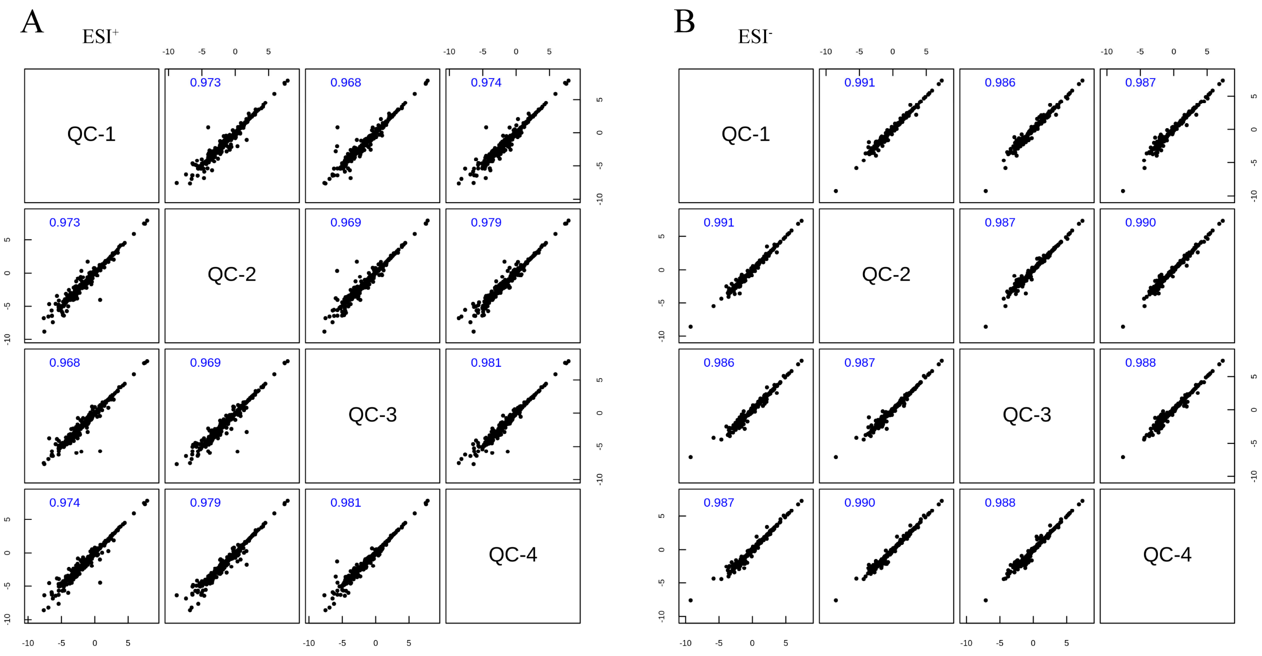

FIGURE S2 Quality control (QC) sample correlation analysis in ESI$^+$ (A) and ESI$^-$ (B) modes.

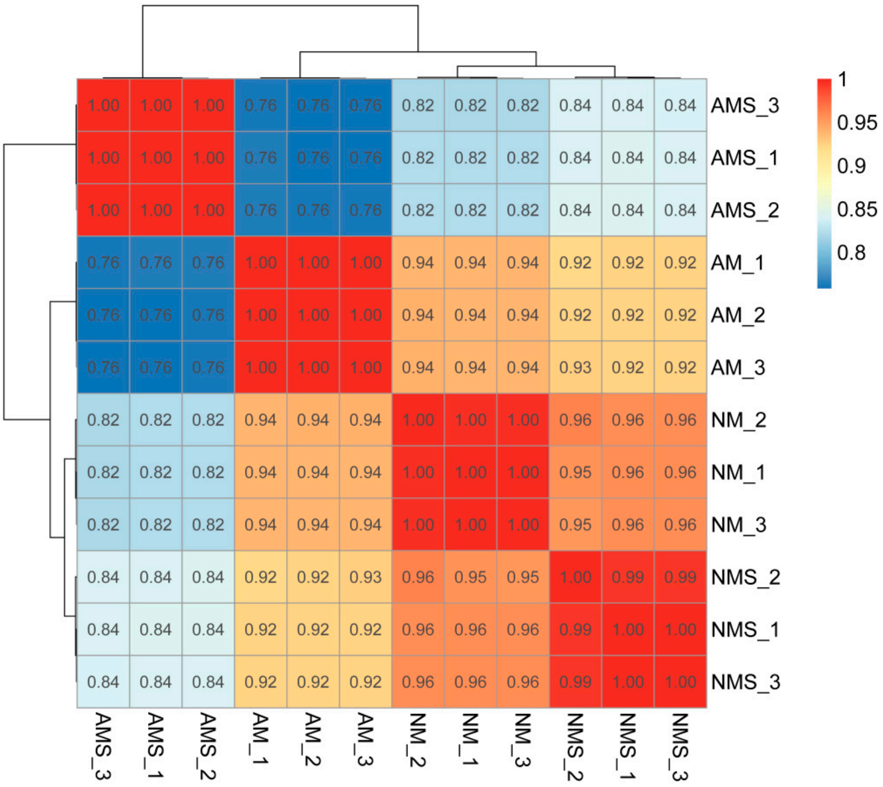

FIGURE S3 Analysis of correlations among different treatment groups.

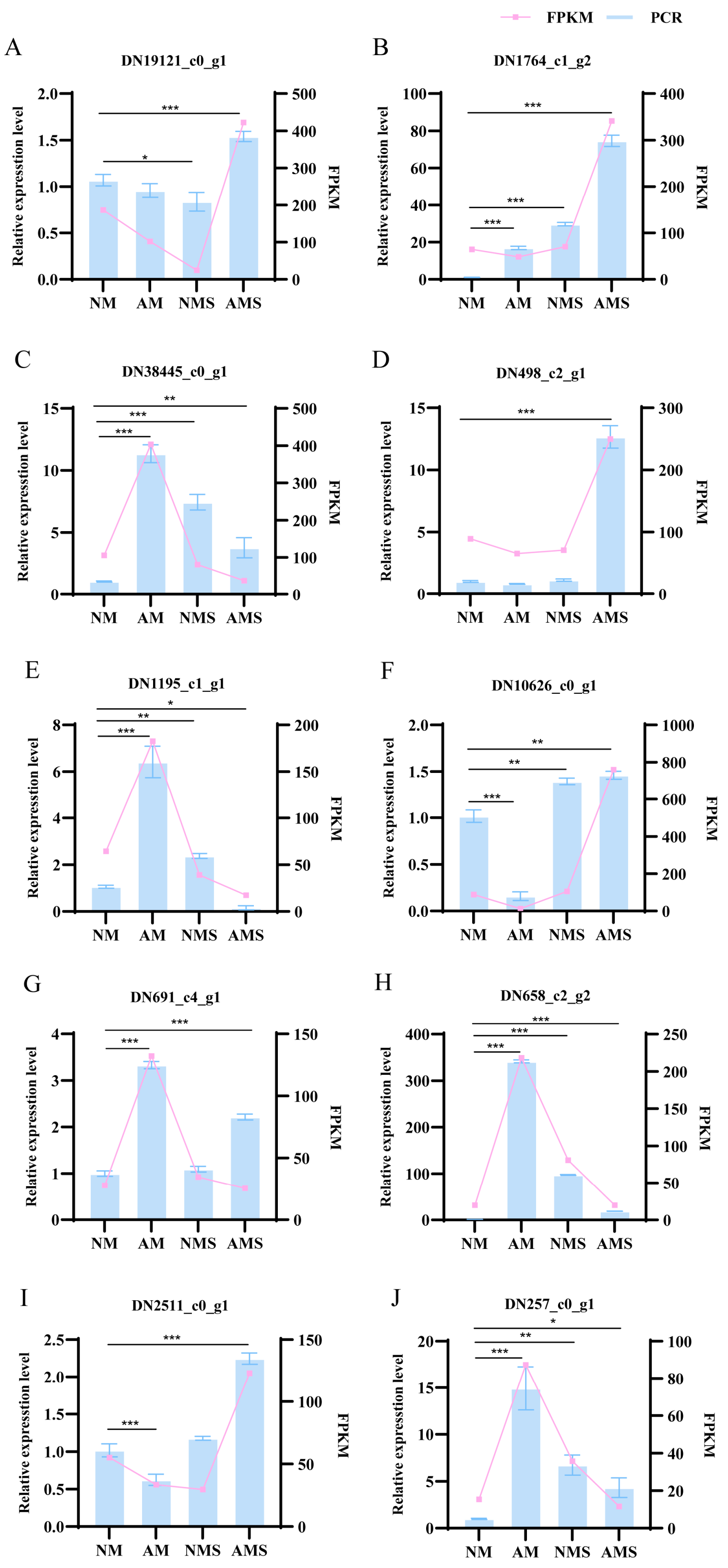

FIGURE S4 (A-J) Transcription levels of 10 DEGs were analyzed by qRT-PCR and RNA-seq (*$p < 0.05$, **$p < 0.01$, ***$p < 0.001$).

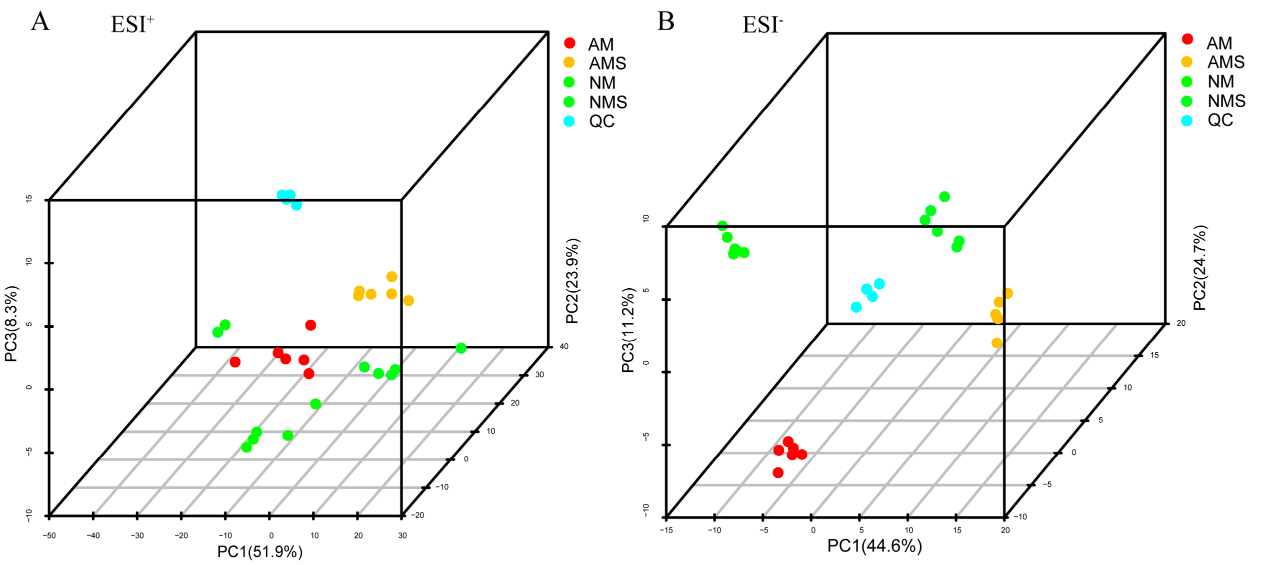

FIGURE S5 The 3D-PCA distribution of different treatment groups (AM, AMS, NM, NMS) and quality control samples (QC) in the ESI$^+$ (C) and ESI$^-$ (D) modes.

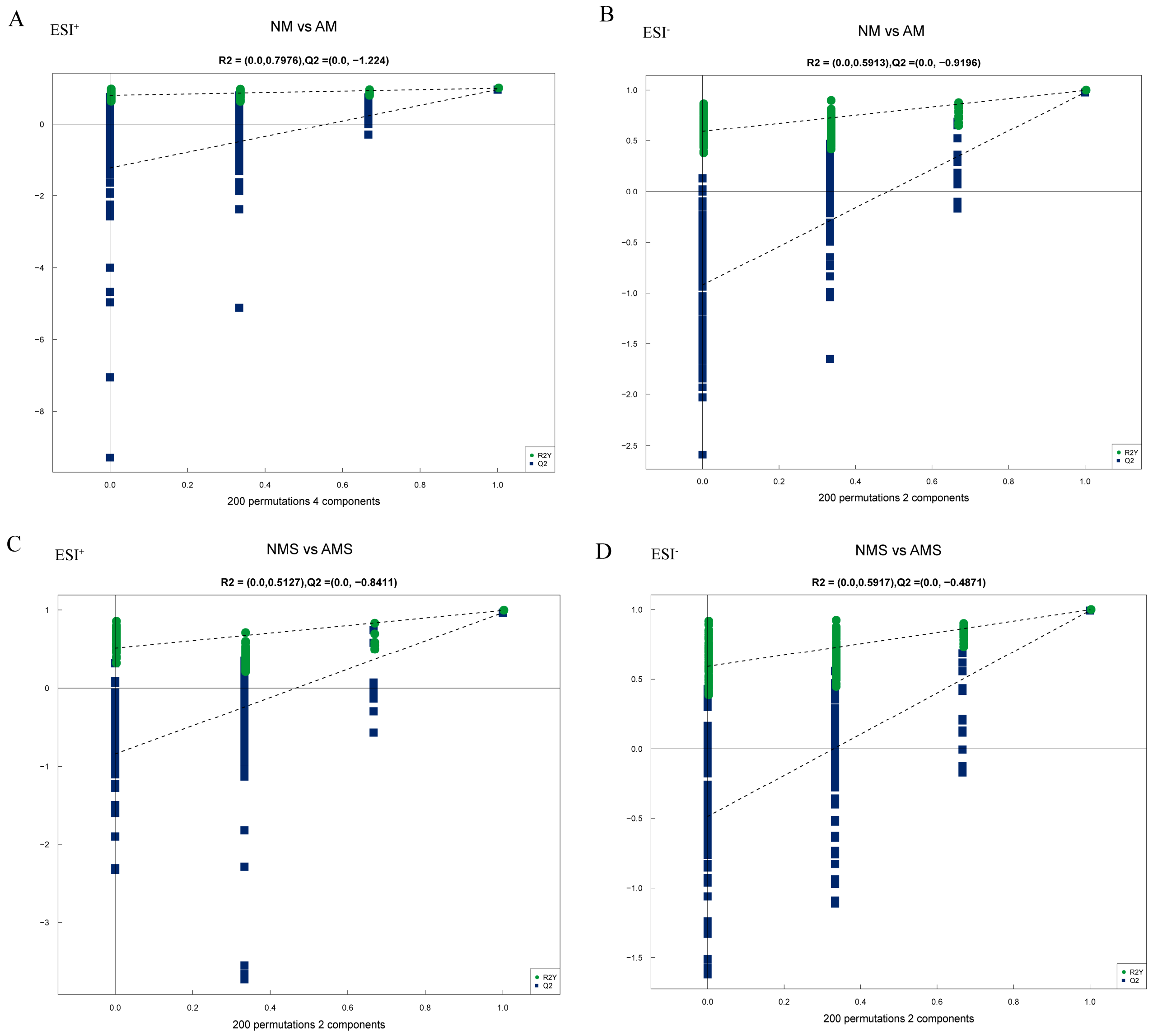

FIGURE S6 PLS-DA permutation tests under different ionization modes. (A-B): NM vs AM; (C-D): NMS vs AMS (positive and negative ion modes, respectively).

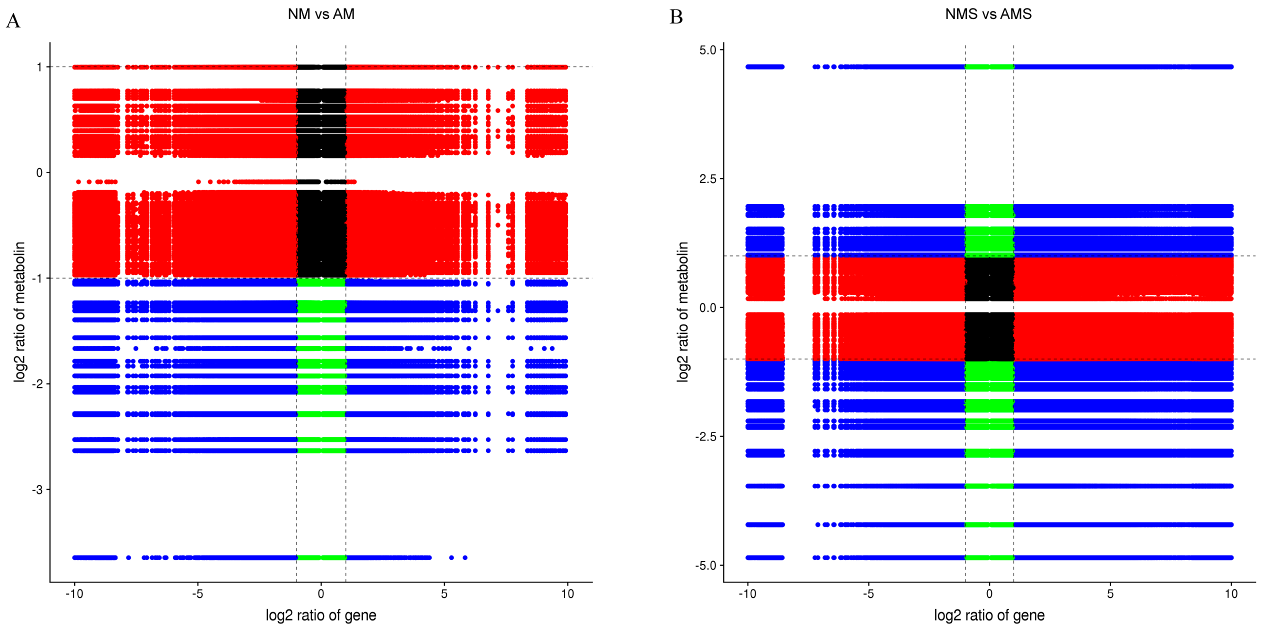

FIGURE S7 Nine-quadrant analysis plots. (A) NM vs AM; (B) NMS vs AMS.

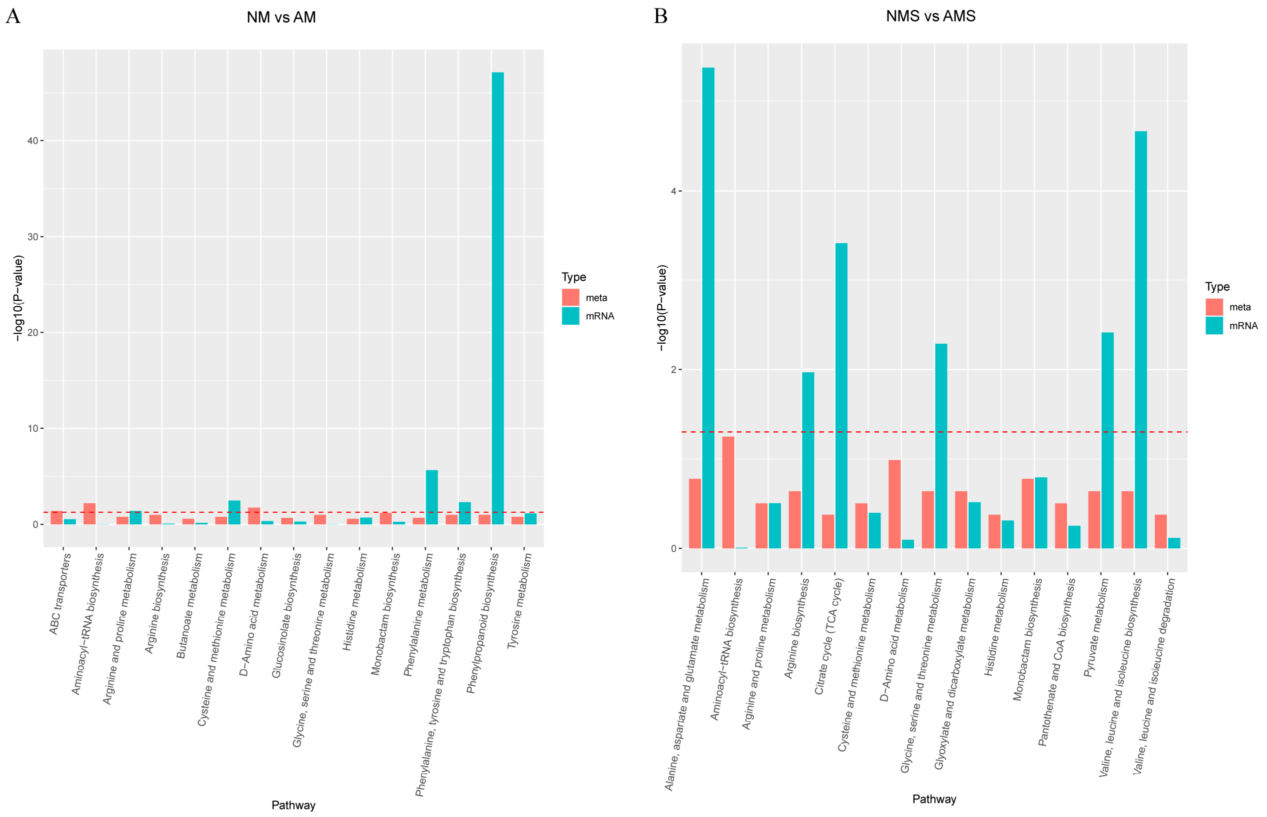

FIGURE S8 KEGG-based correlation analysis of differentially expressed genes (DEGs) and differentially accumulated metabolites (DAMs), (A) NM vs AM; (B) NMS vs AMS.
